# Bone turnover markers are risk factors for endplate injuries during lumbar interbody fusion: a retrospective case-control study

**DOI:** 10.1186/s13018-025-05585-7

**Published:** 2025-02-22

**Authors:** Tae Hoon Kang, Sung Taek Chung, In-Wook Seo, Minjoon Cho, Jae Hyup Lee

**Affiliations:** 1 Present Address: Department of Orthopedic Surgery, SMG-SNU Boramae Medical Center, 20, Boramae-ro 5-gil, Seoul, South Korea; 2 Present Address: Department of Orthopedic Surgery, Seoul National University College of Medicine, 103, Daehak-ro, Jongno-gu, Seoul, Republic of Korea

**Keywords:** Bone turnover marker, Osteoporosis, Bone mineral density, Intraoperative endplate injury, Endplate injury, misTLIF, Lumbar interbody fusion

## Abstract

**Background:**

Intraoperative endplate injury (IEI) is a type of fracture and a potential complication during lumbar interbody fusion (LIF). Osteoporosis diagnosed by bone mineral density (BMD) is a well-known risk factor for fracture itself and IEI also. The bone turnover markers (BTMs) are parameters of bone qualities and have some correlations with fractures, but there is no study about the BTMs and intraoperative fractures especially IEI. This study aims to identify the correlation between IEI and BTMs, especially in misTLIF.

**Methods:**

We retrospectively reviewed 184 patients (230 spine levels). The IEI was diagnosed as the breakage of the endplate observed on postoperative 1 mm thin-cut CT scans. All surgical and endogenous risk factors of IEI were also checked including the bone resorption marker (serum CTX) and bone formation marker (serum P1NP) of BTMs. Additionally, the ratio (P1NP/CTX) and the subtype groups of BTMs were analyzed.

**Results:**

The rate of total IEI was 38%. The sex, osteoporosis, spine BMD, femur BMD, CTX, P1NP/CTX, preoperative disc height, and the discrepancy between preoperative disc height and cage size were risk factors in multivariate logistic regression analyses. The subtypes according to BTMs showed a different rate of IEI, resulting in subtype 2 A (low CTX and P1NP and high P1NP/CTX ratio) having the lowest incidence and statistically significant odds ratios compared to other subtype groups.

**Conclusion:**

This study demonstrated that the IEI is related to BTMs regardless of BMD in misTLIF. In addition, the P1NP/CTX ratio or subtypes could be helpful in predicting the risk of IEI due to the parallel dynamics of BTMs.

## Introduction

A bony fracture is a break in the continuity of a bone, and intraoperative bone fractures are potential complications during surgeries involving bone manipulation. Osteoporosis is a well-known risk factor for both general fractures and intraoperative bone fractures [1]. The gold standard for diagnosing osteoporosis is measuring bone mineral density(BMD) using dual-energy x-ray absorptiometry(DEXA). However, there have been efforts to identify laboratory markers that better reflect bone quality, leading to the emergence of bone turnover markers (BTMs) as potential tools [2]. 

BTMs are classified into two main types. The first is bone formation marker, such as serum P1NP (sP1NP), which reflect the status of bone formation. The second is bone resorption markers, such as serum CTX (sCTX), which indicate the degree of bone resorption. Some studies have explored the ratio between bone formation and resorption markers and even categorized subtypes based on their respective levels [2, 3]. Numerous studies have shown that BTMs are associated with osteoporotic fractures, with some studies demonstrating this correlation independently of BMD [2]. 

Lumbar interbody fusion (LIF) is a procedure that promotes fusion between two vertebrae by removing the intervertebral disc and inserting autologous bone or a bone substitute, often with a cage and pedicle screws for stabilization. Various approaches to LIF include anterior LIF, oblique LIF, posterior LIF, and transforaminal LIF [4]. Transforaminal lumbar interbody fusion (TLIF) involves removing one facet joint and preparing the endplate via the side of the dura mater. With advancements in minimally invasive surgical techniques, minimally invasive TLIF (misTLIF) has become widely adopted [5].

To enhance fusion rates, sufficient removal of disc material and cartilaginous endplates is necessary to expose the bony endplates—a process known as endplate preparation. Additionally, various types of cages are inserted into the intervertebral disc space to support fusion. During these procedures, fractures of the bony endplates, referred to as intraoperative endplate injuries (IEI), may occur. Notably, IEI can also occur during the process of cage insertion [6]. The consequences of IEI include cage subsidence (CS) or cage retropulsion (CR), leading to loss of intervertebral disc height restoration or re-stenosis. These complications can result in neuralgia recurrence, reduced surgical satisfaction, and an increased risk of reoperation [7–9]. 

Several studies have investigated the risk factors for IEI [7, 9]. Osteoporosis remains the most well-known factor, as IEI is a type of fracture inherently linked to bone quality [7, 8, 10–14]. However, surgeons have observed cases of IEI in patients with normal DEXA results, and some studies have further reported no significant correlation between IEI and DEXA measurements [12]. Quantitative computed tomography (qCT) can serve as an alternative approach for more precise bone quality assessment. However, its routine use is limited due to high costs and insurance-related issues [15]. This raises the possibility that poor bone quality, undetectable by DEXA, could be an underlying cause of IEI—potentially explained by BTMs. Unfortunately, no prior studies have examined the relationship between BTMs and intraoperative bone fractures, particularly IEI.

This study aims to analyze the correlation between BTMs and IEI in patients undergoing misTLIF and to determine the odds ratio of various factors, including BTMs, associated with IEI. Our hypothesis is that BTMs are related to the occurrence of IEI, independent of BMD.

## Methods

### Study design and materials

This retrospective case-control study was approved by our Institutional Review Board (IRB) (30-2023-95). Patients who underwent minimally invasive TLIF (misTLIF) at the SMG SNU-Boramae Medical Center Orthopedic Surgery Department between January 2019 and October 2023 were included. All surgeries were performed by two professors, each with over five years of experience in lumbar spine surgery. Cases involving more than three surgical levels or those lacking postoperative CT scans were excluded. A total of 184 patients and 230 intervertebral disc spaces were analyzed (Table 1).

**Table 1:** Patient demographics and surgical parameters

	Patients (*N* = 184)
**Age**	70.53 ± 7.13
**Sex (male/female)**	69/115 (38%/62%)
**Osteoporosis diagnosis by DEXA**	
Normal	45(25%)
Osteopenia	72(39%)
Osteoporosis	67(36%)
**Surgery level**	
1 level	138(75%)
2 levels	46(25%)
**Surgery location**	*N* = 230
L1-2	1
L2-3	3(1%)
L3-4	31(14%)
L4-5	130(57%)
L5-S1	65(28%)
**Disease type by level**	*N* = 235
Spinal stenosis only	65(28%)
Degenerative spondylolisthesis	115(48%)
Isthmic spondylolisthesis	17(7%)
Degenerative retrolisthesis	24(10%)
Degenerative segmental scoliosis	14(6%)
**Cage type**	***N*** = **230**
PEEK	176(77%)
3D printed titanium	54(23%)

### Analysis of risk factors

According to previous studies, the risk factors for intraoperative endplate injury (IEI) can be categorized into surgical factors and endogenous factors related to the patient [7, 8, 10–14]. Surgical factors include the number of surgical levels, preoperative disc height, cage type, cage size, the discrepancy between cage size and disc height, and the surgeon’s proficiency. In this study, we recorded the type of cage (PEEK or 3D-printed titanium), the number of operated levels, surgical level, preoperative disc space height, cage height, and the discrepancy between cage size and disc height. Preoperative disc space heights were measured using preoperative MRI scans (3.0T CX Ingenia, Philips; spacing: 4.4 mm, slice thickness: 4 mm, slice gap: 0.4 mm). Using the 3D-reconstructed mid-sagittal plane, distances between three points (anterior, mid, and posterior) on each upper and lower endplate were measured, and the average value was used [16]. 

Endogenous factors, including patient age, sex, and bone quality-related data, were also analyzed. Preoperative DEXA scans of the lumbar spine and femur were performed according to our hospital protocol, and patients were classified as normal, osteopenic, or osteoporotic using the conventional T-score method. Minimal BMD values for the lumbar spine and femur were recorded. Additionally, blood tests measuring bone metabolism factors, including serum calcium (sCa²⁺), serum phosphorus (sP⁻), and serum parathyroid hormone (sPTH), were performed.

For bone turnover markers (BTMs), serum CTX (sCTX), a bone resorption marker, was measured. About bone formation markers, serum osteocalcin levels were available for 42 patients, while serum P1NP (sP1NP) was measured in 142 patients. Serum CTX and P1NP levels were determined using an electrochemiluminescence immunoassay (ECLIA) performed with Roche’s Cobas analyzer system. The P1NP/CTX ratio was calculated to determine the dominant bone turnover activity. Due to the limited number of patients with osteocalcin data, the analysis primarily focused on the P1NP/CTX ratio.

To classify the BTMs, six subtypes were created based on reference values of sCTX (0.25 µg/L) and sP1NP (32 µg/L). The subtypes were as follows:

- Subtype 1 (sCTX < 0.25, sP1NP > 32): Increased bone formation and decreased resorption.
- Subtype 2 (sCTX < 0.25, sP1NP < 32): Decreased both formation and resorption.
- Subtype 3 (sCTX > 0.25, sP1NP < 32): Decreased formation and increased resorption.
- Subtype 4 (sCTX > 0.25, sP1NP > 32): Increased both formation and resorption.

Subtypes 2 and 4 were further divided based on the P1NP/CTX ratio. Subtype 2A or 4A had a ratio greater than 128, indicating dominance of bone formation, whereas subtype 2B or 4B had a ratio lower than 128, indicating dominance of bone resorption. The incidence of IEI was then analyzed across these subtype groups.

### Diagnostic criteria of intraoperative endplate injury (IEI)

**Fig. 1:**
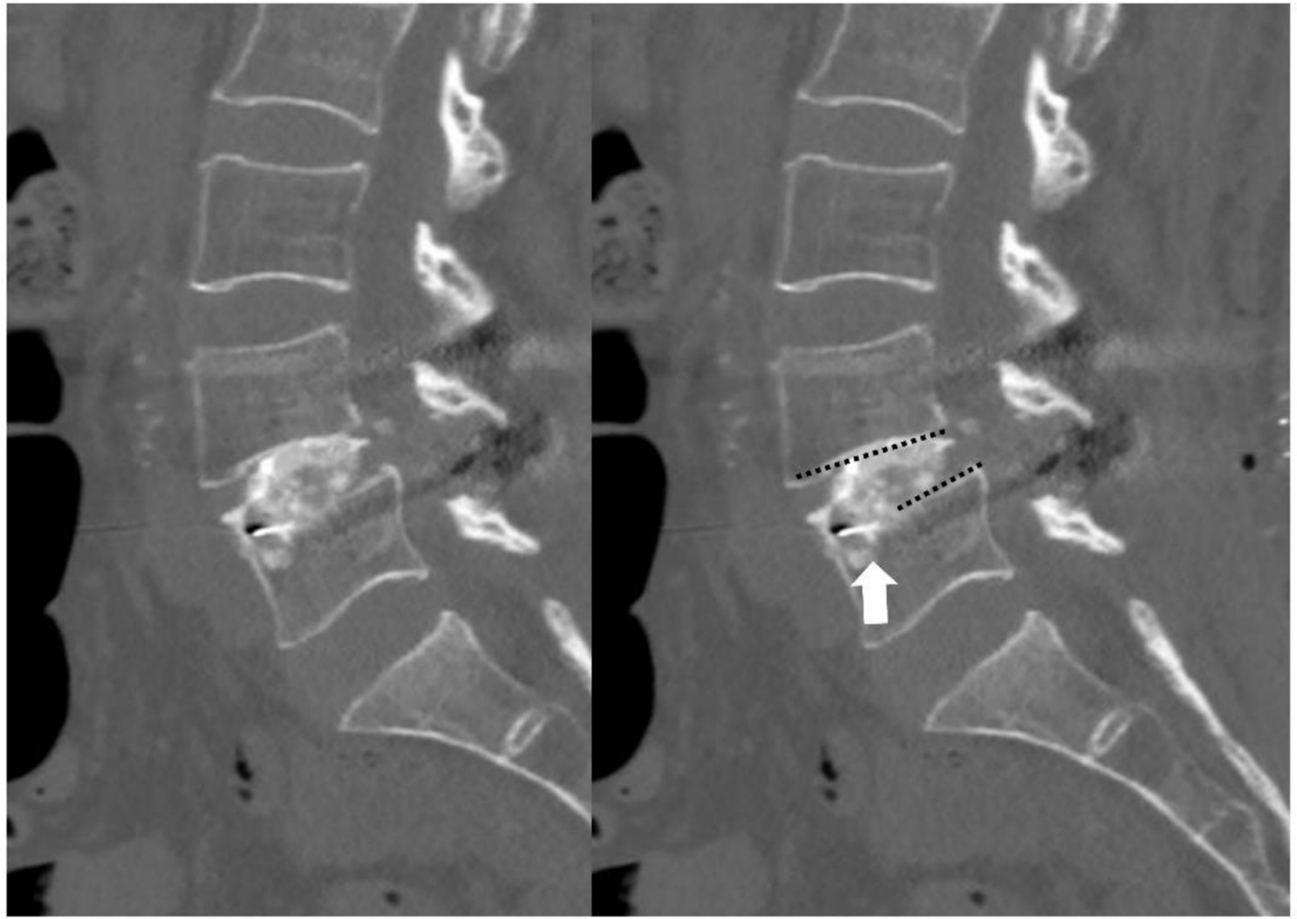
Intraoperative endplate injury on 1mm thin cut reconstructed sagittal scan of postoperative CT, black dotted line - endplate, white arrow - intraoperative endplate injury

One-millimeter thin-cut reconstructed CT scans were performed on postoperative day 2 (Ingenuity, Philips; field of view: 267 × 347, slice thickness: 1 mm, no spacing). The scans were reviewed independently by two spine surgeons, each with over five years of experience in interpreting spinal imaging and diagnosing endplate injuries. To ensure consistency and minimize bias, the reviewers used predefined criteria for diagnosing IEI. The interobserver agreement was high, with a Cohen’s kappa value of 0.92, and any discrepancies were resolved through consensus discussions. (Fig. 1) IEI severity was categorized based on depth: mild for depths less than 1 mm, moderate for depths between 1 and 2 mm, and severe for depths greater than 2 mm.

### Statistical analysis

Statistical analyses were performed to compare variables between the IEI and non-IEI groups. Continuous and non-continuous variables were compared using a two-sample t-test, while categorical variables were analyzed using Fisher’s exact test or chi-square test. The Mann-Whitney U test was used for non-normally distributed continuous variables.

Logistic regression was conducted to identify factors associated with IEI. Variables with *P* < 0.05 in the univariate analysis were included in the multivariate logistic regression, with sequential variable selection to account for correlations. Multivariate logistic regression was also used to analyze odds ratios among subtype groups.

All statistical analyses were performed using SPSS version 16.0 (Chicago, Illinois), and a P-value of less than 0.05 was considered statistically significant.

## Results

### Incidence of IEI

IEI occurred in 74 out of 184 patients (40.2%). In terms of surgical levels, IEI occurred in 86 out of 230 levels (37.4%), with the majority being of moderate severity (64%). The intergroup consistency comparison between the two surgeon groups showed similar IEI incidence rates per level (35.2% vs. 39.8%, *p* = 0.475). As no significant difference was observed, the groups were not analyzed separately. The characteristics of the 86 levels with endplate injuries are outlined in Table 2.

**Table 2:** The characteristics of the intraoperative endplate injuries

	Endplate injury level(*N* = 86/230)
Upper	33(38%)
Lower	21(24%)
Both level	32(37%)
L1-2	0
L2-3	1
L3-4	12(14%)
L4-5	43(50%)
L5-S1	30(35%)
**Definition of injury**	
Mild (within 1 mm)	26(30%)
Moderate (1 ~ 2 mm)	55(64%)
Severe (over 2 mm)	5(6%)

### Comparison of risk factors between IEI and Non-IEI groups

All risk factors of IEI were compared between the IEI and non-IEI groups. (Table 3)

**Table 3:** Risk factors between the endplate injury and non-endplate injury groups

			Endplate injury(*N* = 74)		Non-endplate injury(*N* = 110)	*p*-value
**Age**			70.68 ± 7.12		70.44 ± 7.20	0.828*
**Sex (male/female)**			17/57(23%/77%)		52/58(46%/54%)	**0.002†**
**Osteoporosis diagnosis**						**< 0.001†**
Normal			14(19%)		31(28%)	
Osteopenia			20(27%)		52(47%)	
Osteoporosis			40(54%)		27(25%)	
**Surgery level**						0.056†
1 level			50(68%)		88(80%)	
2 levels			24(32%)		22(20%)	
**Disease type**			*N* = 90		*N* = 145	0.235†
Spinal stenosis only			28(31%)		37(26%)	
Degenerative spondylolisthesis			41(46%)		74(51%)	
Isthmic spondylolisthesis			5(6%)		12(8%)	
Degenerative retrolisthesis			9(10%)		15(10%)	
Degenerative segmental scoliosis			7(8%)		7(5%)	
**Cage type**			*N* = 86		*N* = 144	0.481†
PEEK			68(79%)		108(75%)	
3D printed titanium			18(21%)		36(25%)	
**Surgical factors**			*N* = 86		*N* = 144	
Pre-operative disc height (mm)			8.82(7.27–9.31)		9.31(8.52–10.32)	**< 0.001‡**
Post-operatvie disc height (mm)			10.88 ± 1.57		11.24 ± 1.82	0.093*
Cage size (mm)			1(8)/11(9)/39(10)/24(11)/9(12)/2(13)		7(8)/20(9)/46(10)/57(11)/14(12)/0(13)	0.069**†**
Discrepancy between preoperative disc height and cage size			1.92(1.03–2.97)		0.87(0.24–1.71)	**< 0.001‡**
**DEXA**						
Spine BMD (g/cm^2)			0.887(0.792–1.070)		1.014(0.891–1.169)	**0.003‡**
Femur BMD (g/cm^2)			0.738(0.647–0.831)		0.795(0.732–0.924)	**0.002‡**
**Endogenous factors**	normal range					
sCa^2+^ (mg/dL)	8.8 ~ 10.5(mg/dL)		9.2(8.9–9.5)		9.3(9.0–9.53)	0.174‡
sP^−^ (mg/dL)	2.5 ~ 4.5(mg/dL)		3.6(3.3–3.8)		3.5(3.2–3.9)	0.65‡
Vitamine D (25-(OH) vit.D) (ng/mL)	30.1 ~ 100 (ng/mL)		28.2(16.1–36.1)		28.1(18–37.9)	0.693‡
PTH (pg/ml)	10 ~ 65 (pg/ml)		31(17–45.5)		22(13–37.5)	0.056‡
**Bone Turnover Marker**						
sCTX (ng/mL)	0.025 ~ 0.573 (ng/mL)		0.389(0.239–0.602)		0.275(0.182–0.381)	**< 0.001‡**
			*N* = 19		*N* = 23	
Osteocalcin (ng/mL)	male: 10.00 ~ 46.00 (ng/mL)		7.7(4.80–16.92)		13.3(6.70–15.52)	0.622‡
	female: 10.00 ~ 46.00 (ng/mL)					
			*N* = 54		*N* = 85	
sP1NP (ng/mL)	male: 22.90 ~ 85.30 (ng/mL)		48.8(26.98–68.23)		35.9(26.86–62.00)	0.121‡
	female: 16.27 ~ 73.87 (ng/mL)					
			*N* = 54		*N* = 85	
P1NP/CTX			107.59(83.57–180.46)		155.31(121.25–201.93)	**0.001‡**
**Subtype (P1NP/CTX)**			*N* = 54		*N* = 85	**0.029†**
1			5(9%)		13(18%)	
2a			2(4%)		18(24%)	
2b			8(15%)		10(14%)	
3			6(11%)		7(9%)	
4a			16(30%)		24(32%)	
4b			17(31%)		13(18%)	

*two-sample test

†Chi-square test

‡Mann Whitney U-test

Females and osteoporotic patients were more predominant in the IEI group. While there was a tendency for IEI in two-level surgeries, the p-value was 0.056. There was no difference in disease type, surgical location, or cage types. Lower preoperative disc space height and larger discrepancy between preoperative disc space height and cage size were related to IEI. Absolute BMD scores of the lumbar spine and femur were lower in the IEI group. Notably, sCTX levels were significantly higher in the IEI group but osteocalcin and sP1NP showed no correlation. The P1NP/CTX ratios were lower in the IEI group. There was a difference in the distribution of subtypes between two groups.

About the treatment of osteoporosis, only 11 of 40 osteoporotic patients in the IEI group were under treatment. In the non-IEI group, 4 out of 27 were under treatment. Statistically, there was no significant difference between the two groups. (Table 4)

**Table 4:** Osteoporosis treatment in two groups

	Total osteoporosis	Endplate injury	Non-endplate injury	
Osteoporosis treatment	15/67 (22.4%)	11/40 (27.5%)	4/27 (14%)	0.222†
Osteoporosis treatment per level	16/80 (20%)	11/45 (24.4%)	5/35 (14.3%)	0.260†

†Chi-square test

## Multivariate logistic regression analysis

Multiple logistic regression analysis was performed to analyze the relationship between IEI occurrence and several independent variables. Before it, univariate logistic regressions were conducted. Statistically correlated variables included sex, osteoporosis diagnosis, spine BMD scores, femur BMD scores, sCTX, P1NP/CTX ratio, preoperative disc height, and the discrepancy between preoperative disc height and cage size. Multivariate logistic regression was performed after eliminating interference effects. As a result, three major categories were independently related to endplate injury: osteoporosis-related factors (sex, presence of osteoporosis, BMD scores), BTMs (CTX, P1NP/CTX), and surgical factors (preoperative disc height, discrepancy between preoperative disc height and cage size) (Table 5).

**Table 5:** Univariate and multivariate analyses of intraoperative endplate injury

	Univariate analysis						Multivariate analysis									
	OR	95% CI			*p* value						OR		95% CI			*p* value
sex (female)	2.715	1.493–4.939			**0.001**						2.393		1.289–4.446			0.006*
age	1.003	0.967–1.041			0.865											
fusion level (2 levels)	1.131	0.657–1.949			0.656											
osteopenia	0.898	0.432–1.868			0.774											
osteoporosis	3.176	1.552–6.499			**0.002**						3.539		1.649–7.598			0.001*
Spine BMD (x10^3)	0.997	0.996–0.999			**0.001**						0.997		0.995–0.999			< 0.001*
femur BMD (x10^3)	0.997	0.995–0.999			**0.002**						0.997		0.994–0.999			0.010*
Ca	0.759	0.437–1.317			0.326											
P	1.171	0.715–1.919			0.530											
vit.D	0.992	0.974–1.009			0.355											
PTH	1.008	0.997–1.018			0.142											
osteocalcin	0.988	0.904–1.079			0.784											
P1NP	1.007	0.999–1.016			0.100											
CTX (x10^3)	1.003	1.001–1.004			**< 0.001**						1.003		1.002–1.005			**< 0.001**
P1NP/CTX	0.995	0.991–0.999			**0.019**						0.994		0.990–0.999			**0.015†**
preoperative disc height	0.689	0.576–0.824			**< 0.001**						0.698		0.551–0.885			**0.003**
Discrepancy between preoperative disc height and cage size	1.754	1.401–2.196			**< 0.001**						1.624		1.230–2.143			**0.004**

* Adjusted between sex, osteoporosis, BMD

†Adjusted between CTX and P1NP/CTX

### Subtype analysis and odds ratios

**Fig. 2:**
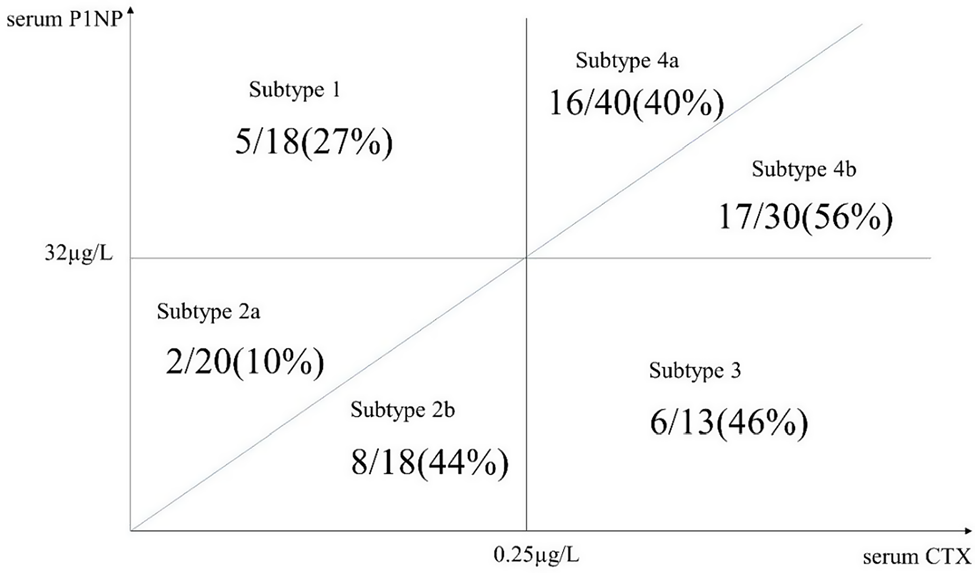
The incidence rate of intraoperative endplate injury of each subtype

The lowest incidence was observed in group 2 A (10%). The highest incidence was seen in group 4B (56%). (Fig. 2)

Odds ratios (OR) of IEI between each of the six groups were analyzed. According to simple logistic regression analyses, ORs between subtype 2 A and others (2B, 3, 4 A, and 4B groups) showed a significant difference. As known, subtypes were related to osteoporosis factors, ORs were recalculated through multiple logistic regression tests, excluding the effects of sex, BMD, and osteoporosis. As a result, the ORs between subtype 2 A and others (2B, 3, 4 A, and 4B groups) remained statistically significant (Table 6).

**Table 6:** Univariate and multivariate analyses between subtypes of intraoperative endplate injuries

Subtype	Univariate analysis			Multivariate analysis*		
	OR	95% CI	*p* value	OR	95% CI	*p* value
1	(reference)					
2a	0.289	0.048–1.727	0.174			
2b	2.340	0.595–9.202	0.224			
3	2.275	0.535–9.666	0.265			
4a	1.842	0.552–6.140	0.320			
4b	3.400	0.965–11.975	0.057	3.687	0.891–15.255	0.072
2a	(reference)					
2b	8.100	1.456–45.060	**0.017**	16.040	1.608–160.051	**0.018**
3	7.875	1.330–46.628	**0.023**	16.287	1.552–170.968	**0.020**
4a	6.375	1.303–31.184	**0.022**	16.923	1.876–152.681	**0.012**
4b	11.769	2.307–60.045	**0.003**	29.045	3.052–276.387	**0.003**
2b	(reference)					
3	0.972	0.250–3.775	0.968	1.008	0.223–4.551	0.992
4a	0.787	0.264–2.350	0.668	1.044	0.296–3.685	0.947
4b	1.453	0.458–4.609	0.526	1.806	0.468–6.979	0.391
3	(reference)					
4a	0.81	0.246–2.660	0.728	1.062	0.285–3.950	0.929
4b	1.495	0.430–5.191	0.527	1.958	0.466–8.229	0.359
4a	(reference)					
4b	1.846	0.712–4.786	0.207	1.878	0.596–5.921	0.282

* Excluding the effect of sex, spineBMD, femurBMD, osteoporosis

## Discussion

This study aimed to analyze the correlation between a kind of intraoperative fractures, IEI, and BTMs. Although several studies have examined the relationship between fractures and BTMs, none have focused on intraoperative bone fractures. This was the first investigation into the connection between intraoperative bone fractures and BTMs, related to the bone quality independently of BMD. It can provide valuable insights for orthopedic and spine surgeons who works with bones, to minimize the risk of intraoperative bone fractures and improve the overall quality of operations. As concerned, spine surgeons are vigilant against intraoperative endplate injuries. We experienced that there are some patients with poor bone quality, but the relatively normal value of BMD. There was no way to predict this situation [17]. Although advanced imaging techniques like high-resolution CT and peripheral QCT can assess bone microarchitecture, their routine use is limited by cost, radiation exposure, and availability, with some reports also questioning the effectiveness of lumbar spine CT HU measurements as a screening tool for low BMD [17–20]. In this situation, this study can address the gap between the real bone quality and BMD, suggesting that BTMs could offer a valuable explanation. Preoperative assessment of BTMs could be crucial in avoiding intraoperative fractures, aiding surgeons in formulating strategies to deal with bone-related challenges, irrespective of BMD.

In our study, we chose to review the mis TLIFs because there are various risks of IEI for different types of LIFs and the mis TLIFs have a tendency of higher rate due to small working space for minimal invasiveness [21]. And a higher rate of IEI could present more study numbers of IEI to be analyzed. In our data, the incidence of IEI per level was 37.4%, higher than in other previous studies of LIFs [10]. This elevated incidence is due to our 1 mm thin-cut CT scans, in contrast to studies relying on X-ray images or routine CT scans and we counted even minor damage [10, 14]. According to previous studies, IEI does not necessarily lead to cage problems like CS, and even if CS occurs and IEI itself were separate from clinical symptoms [22]. 

As concerned, we contained well-known risk factors for IEI. Notably, the cage material was unrelated to IEI, while the preoperative disc height and the discrepancy between disc height and cage size were not. Because the low preoperative disc height could lead to IEI due to limited working space. Additionally, using a larger cage to small disc space to restore the collapsed disc space could result in IEI during the cage insertion [12]. 

BTMs have been studied as a way to evaluate the degree of bone metabolism biochemically. The BTMs can objectively reflect the turnover rate of the bone microarchitecture [2]. In our data, sCTX was one of main risk factor for IEI regardless to BMD. CTX is a C-terminal telopeptide of type 1 collagen produced during bone microstructure resorption, reflecting bone resorption mechanisms and microstructure deterioration. Elevated sCTX levels indicate high bone microarchitecture resorption and this status was vulnerable to some impacts or maneuvers to bony structures, as evidenced by our data. However, sP1NP and osteocalcin, the bone formation markers, were not relative to IEI. The P1NP is N-terminal propeptide of type 1 collagen made during the bone microstructure formation and the osteocalcin is produced by osteoblasts, the bone forming cells. We concluded that it is related with “the parallel dynamic” of BTMs’ natural characterictics [3]. During the bone remodeling, the continuous metabolism of living skeletons, the rapid bone turnover means rapid resorption and rapid re-formation of microstructures. This causes both increases of bone formation and resorption markers [3]. Considering the parallel dynamic, the concept of P1NP/CTX ratio was applied. The odd ratio of IEI was less than 1 because it means more resorption than formation of bony microstructure beyond the speed of the bone turnover itself. This was proved by multivariate analysis too, which means regardless of BMD and surgical related factors.

Furthermore, we applied the subtypes in terms of sP1NP and sCTX. In the previous study, the normal state was considered as subtype 1 [3]. In our study, although there is no statistically difference between subtype 1 and 2 A, the subtype 2 A showed the lowest incidence of IEI. This must be related to the parallel dynamic too. The subtype 2 A group has lower P1NP than the subtype 1 and this could mean lower turnovers of bone microstructure. In the same way, the subtype 4B showed higher incidence of IEI than group 3. Theoretically, the subtype 3 should show the highest incidence rate but according to the parallel dynamics, in the subtype 4B, both formation and resorption markers increased and the ratio decreased, which resulted in highest turnovers. In short, in our results, the safest subtype from fractures was subtype 2 A, confirmed by the multiple logistic regression adjusted other factors. The subtype system could be helpful to analyze the risk of IEI.

In our data, there was no correlation between the osteoporosis treatment and risk of fracture. This could be due to multiple reasons. First, there are so many types of osteoporosis treatment. It is impossible to combine several types into one. Second, so many patients didn’t know they had osteoporosis or not. Also, many patients had difficulty in remembering the exact treatment. More detailed study about the osteoporosis medication might be helpful about it.

Based on the results, the 4B group exhibited the highest risk of IEI, necessitating particular caution during surgery. Specifically, if the surgery is not emergent, adjusting the timing of the procedure and optimizing bone quality prior to surgery could be beneficial. Although the current study did not find a significant correlation between osteoporosis treatment and IEI, this is likely due to the aforementioned limitations. Previous studies have shown that the use of antiresorptive or anabolic agents can improve not only DEXA scores but also BTMs [23]. Therefore, in elective surgeries, the preoperative use of these medications could help mitigate IEI risk. In terms of modifiable surgical factors, selecting a cage size that is appropriate but relatively smaller could help minimize the risk of IEI. However, overly small cages may limit the improvement of foraminal height. To address this, sufficient laminectomy or facetectomy should be performed to maximize the surgical outcome, even when using a relatively small cage [24, 25]. These strategies could be instrumental in reducing IEI risk while maintaining the overall efficacy of the procedure.

The biggest drawback of the BTM is the difficulty of standardization. BTMs change throughout the day. Some fasting or checking laboratory test in a planned time is needed [2, 3, 26]. Fortunately, the study has consistent data because the tests were performed preoperatively, after fasting, and in the same institution. But different outcomes may occur in different situations.

The limitation of this study is that first, the number of patients is not large. It is because the BTMs are started to be checked recently due to insurance coverage policies of our country. Second, this is a retrospective study. More detailed prospective study might be needed. Third, this study does not cover long-term union rates or complications like cage subsidence. Instead, we focused on the immediate occurrence of IEI, which can be irrelevant to bone unions or clinical prognoses.

## Conclusion

This study demonstrated that the intraoperative endplate injury, one of intraoperative bone fractures, was related to bone turnover markers regardless of bone mineral density, particularly during minimally invasive TLIFs. Considering the parallel dynamics, the P1NP/CTX ratio or subtypes could be helpful. Analyzing bone turnover markers can be a novel strategy to reduce endplate injuries in spine surgeries.

## Data Availability

No datasets were generated or analysed during the current study.

## Abbreviations

BTM: Bone turnover marker
BMD: Bone mineral density
IEI: Intraoperative endplate injury
LIF: Lumbar interbody fusion
TLIF: Transforaminal LIF
misTLIF: minimal invasive TLIF
CS: Cage subsidence
CR: Cage retropulsion
DEXA: Dual-energy x-ray absorptiometry
Ca^2+^: Calcium ion
P^−^: Phosphorous ion
PTH: Parathyroid hormone
CTX: C-terminal telopeptide of type 1 collagen
P1NP: N-terminal propeptide of type 1 collagen

## References

[1] Strømsøe K. Fracture fixation problems in osteoporosis. Injury. 2004;35(2):107–13. 14736465 10.1016/j.injury.2003.08.019

[2] Shetty S, Kapoor N, Bondu JD, Thomas N, Paul TV. Bone turnover markers: emerging tool in the management of osteoporosis. Indian J Endocrinol Metab. 2016;20(6):846–52. 27867890 10.4103/2230-8210.192914 PMC5105571

[3] Fisher A, Fisher L, Srikusalanukul W, Smith PN. Bone turnover status: classification model and clinical implications. Int J Med Sci. 2018;15(4):323–38. 29511368 10.7150/ijms.22747 PMC5835703

[4] Verma R, Virk S, Qureshi S. Interbody fusions in the lumbar spine: a review. HSS Journal^®^. 2020;16(2):162–7. 32523484 10.1007/s11420-019-09737-4 PMC7253570

[5] Park S-M, Kim H-J, Yeom JS. Is minimally invasive surgery a game changer in spinal surgery? Asian Spine J. 2024;18(5):743–52. 10.31616/asj.2024.0337 PMC11538812 39434232

[6] Polikeit A, Ferguson SJ, Nolte LP, Orr TE. The importance of the endplate for interbody cages in the lumbar spine. Eur Spine J. 2003;12(6):556–61. 12783287 10.1007/s00586-003-0556-5 PMC3467986

[7] Shi H, Wang XH, Zhu L, Chen L, Jiang ZL, Wu XT. Intraoperative Endplate Injury following transforaminal lumbar Interbody Fusion. World Neurosurg. 2022;168:e110–8. 36122858 10.1016/j.wneu.2022.09.055

[8] Satake K, Kanemura T, Nakashima H, Yamaguchi H, Segi N, Ouchida J. Cage subsidence in lateral interbody fusion with transpsoas approach: intraoperative endplate injury or late-onset settling. Spine Surg Relat Res. 2017;1(4):203–10. 31440635 10.22603/ssrr.1.2017-0004 PMC6698569

[9] Zhou ZJ, Xia P, Zhao FD, Fang XQ, Fan SW, Zhang JF. Endplate injury as a risk factor for cage retropulsion following transforaminal lumbar interbody fusion: an analysis of 1052 cases. Med (Baltim). 2021;100(5):e24005. 10.1097/MD.0000000000024005 PMC7870182 33592856

[10] Satake K, Kanemura T, Yamaguchi H, Segi N, Ouchida J. Predisposing factors for Intraoperative Endplate Injury of Extreme lateral Interbody Fusion. Asian Spine J. 2016;10(5):907–14. 27790319 10.4184/asj.2016.10.5.907 PMC5081326

[11] Park MK, Kim KT, Bang WS, Cho DC, Sung JK, Lee YS, et al. Risk factors for cage migration and cage retropulsion following transforaminal lumbar interbody fusion. Spine J. 2019;19(3):437–47. 30142459 10.1016/j.spinee.2018.08.007

[12] Kim YH, Ha KY, Kim KT, Chang DG, Park HY, Yoon EJ, et al. Risk factors for intraoperative endplate injury during minimally-invasive lateral lumbar interbody fusion. Sci Rep. 2021;11(1):20149. 34635757 10.1038/s41598-021-99751-6 PMC8505407

[13] Wu H, Shan Z, Zhao F, Cheung JPY. Poor bone quality, multilevel surgery, and narrow and tall cages are Associated with Intraoperative Endplate Injuries and late-onset cage subsidence in lateral lumbar Interbody Fusion: a systematic review. Clin Orthop Relat Res. 2022;480(1):163–88. 34324459 10.1097/CORR.0000000000001915 PMC8673985

[14] Jing X, Gong Z, Zhang N, Xu Z, Qiu X, Li F, et al. Comparison of Intraoperative Endplate Injury between Mini-open lateral lumbar Interbody Fusion (LLIF) and transforaminal lumbar Interbody Fusion (TLIF) and analysis of risk factors: a retrospective study. J Invest Surg. 2023;36(1):2285787. 38010393 10.1080/08941939.2023.2285787

[15] Huang Y, Chen Q, Liu L, Feng G. Vertebral bone quality score to predict cage subsidence following oblique lumbar interbody fusion. J Orthop Surg Res. 2023;18(1):258. 36991489 10.1186/s13018-023-03729-1 PMC10061981

[16] Bach K, Ford J, Foley R, Januszewski J, Murtagh R, Decker S, et al. Morphometric Analysis of Lumbar Intervertebral disc height: an imaging study. World Neurosurg. 2019;124:e106–18. 10.1016/j.wneu.2018.12.014 30579030

[17] Paz RD, Henríquez MS, Melián KA, Martin CB. Prevalence of poor bone quality in patients undergoing spine surgery: a Comprehensive Approach. Global Spine J. 2022;12(7):1412–9. 33487013 10.1177/2192568221989684 PMC9393977

[18] Oei L, Koromani F, Rivadeneira F, Zillikens MC, Oei EH. Quantitative imaging methods in osteoporosis. Quant Imaging Med Surg. 2016;6(6):680–98. 28090446 10.21037/qims.2016.12.13 PMC5219969

[19] Zaidi Q, Danisa OA, Cheng W. Measurement Techniques and Utility of Hounsfield Unit values for Assessment of Bone Quality prior to spinal instrumentation: a review of current literature. Spine (Phila Pa 1976). 2019;44(4):E239–44. 30063528 10.1097/BRS.0000000000002813

[20] Kohan EM, Nemani VM, Hershman S, Kang DG, Kelly MP. Lumbar computed tomography scans are not appropriate surrogates for bone mineral density scans in primary adult spinal deformity. Neurosurg Focus. 2017;43(6):E4. 29191096 10.3171/2017.9.FOCUS17476

[21] Rihn JA, Gandhi SD, Sheehan P, Vaccaro AR, Hilibrand AS, Albert TJ, et al. Disc space preparation in transforaminal lumbar interbody fusion: a comparison of minimally invasive and open approaches. Clin Orthop Relat Res. 2014;472(6):1800–5. 24522382 10.1007/s11999-014-3479-z PMC4016455

[22] Oh KW, Lee JH, Lee JH, Lee DY, Shim HJ. The correlation between cage subsidence, bone Mineral Density, and clinical results in posterior lumbar Interbody Fusion. Clin Spine Surg. 2017;30(6):E683–9. 28632554 10.1097/BSD.0000000000000315

[23] Seeman E, Martin TJ. Antiresorptive and anabolic agents in the prevention and reversal of bone fragility. Nat Rev Rheumatol. 2019;15(4):225–36. 30755735 10.1038/s41584-019-0172-3

[24] Gelfand Y, Benton J, De la Garza-Ramos R, Yanamadala V, Yassari R, Kinon MD. Effect of cage type on short-term Radiographic outcomes in Transforaminal lumbar Interbody Fusion. World Neurosurg. 2020;141:e953–8. 32565381 10.1016/j.wneu.2020.06.096

[25] Kang TH, Cho M, Lee JH. Biportal Endoscopic TLIF with an expandable cage: technical note and preliminary results in terms of Segmental Lordosis Achievement and Disc Height Elevation. Int J Spine Surg. 2024;18(5):571–81. 39500593 10.14444/8680 PMC11616418

[26] Vasikaran S, Eastell R, Bruyere O, Foldes AJ, Garnero P, Griesmacher A, et al. Markers of bone turnover for the prediction of fracture risk and monitoring of osteoporosis treatment: a need for international reference standards. Osteoporos Int. 2011;22(2):391–420. 21184054 10.1007/s00198-010-1501-1

